# The Use of Implantable Cardioverter-defibrillators in the Prevention of Sudden Cardiac Death: A Focus on Congenital Heart Disease and Inherited Arrhythmia Syndromes

**DOI:** 10.19102/icrm.2018.090103

**Published:** 2018-01-15

**Authors:** Sarah A. Goldstein, Cary C. Ward, Sana M. Al-Khatib

**Affiliations:** ^1^Division of Cardiology, Duke University Hospital, Durham, NC, USA; ^2^Duke Clinical Research Institute, Duke University Hospital, Durham, NC, USA

**Keywords:** Congenital heart disease, implantable cardioverter-defibrillator, inherited arrhythmia syndrome, sudden cardiac death, ventricular arrhythmia

## Abstract

Some congenital heart diseases (CHDs) and inherited arrhythmia syndromes are associated with an increased risk of sudden cardiac death (SCD). Appropriate selection criteria for implantable cardioverter-defibrillator (ICD) implantation in these patients are poorly defined due to a paucity of data available from randomized clinical trials, leading to current guidelines relying more on non-randomized studies and expert opinions to make their recommendations. This review describes available evidence-based risk stratification methods for identifying patients at risk for SCD, as well as current guideline-driven management strategies for the use of ICDs in patients with CHD and inherited arrhythmia syndromes.

## Introduction

The occurrence of sudden cardiac death (SCD), primarily due to malignant ventricular arrhythmias (VAs), affects approximately 350,000 people in the United States each year.^1^ Randomized controlled trials have shown that the use of implantable cardioverter-defibrillators (ICDs) can decrease the risk of SCD in the settings of both ischemic and non-ischemic cardiomyopathy.^2,3^ Evidence-based recommendations guiding the use of ICDs for both primary and secondary prevention purposes in these populations are well defined.^4^ With the exception of ischemic heart disease, inherited arrhythmia syndromes are the most common conditions predisposing patients to SCD. Furthermore, the survival of patients with congenital heart disease (CHD) is increasing because medical and surgical care received during childhood has improved, resulting in an additional cohort at risk for malignant arrhythmias. SCD accounts for 20% to 25% of mortality in the adult CHD population.^5–7^

ICDs are able to successfully treat life-threatening VAs in patients with adult CHD and inherited arrhythmia syndromes.^8–10^ However, ICD use in this generally younger patient population is complicated, with high rates of device-related complications occurring over many decades of use, including inappropriate shocks, device-related infections, and lead displacement or failure.^11^ Furthermore, appropriate selection criteria for ICD implantation in these patients are poorly defined due to a paucity of randomized controlled trials in such patients. Current professional guidelines therefore rely on data from non-randomized studies and on expert opinion.^11^ This review describes available evidence-based risk stratification for SCD and current guideline-driven management strategies for the use of ICDs in patients with CHD and inherited arrhythmia syndromes.

## Congenital heart disease

With better surgical outcomes and improved medical management, the CHD population has grown significantly over the past three to four decades, with 85% to 90% of affected children now expected to live into adulthood.^12^ SCD, primarily due to malignant VAs occurring during the third to fifth decade of life, is a leading cause of death in adult patients with CHD.^5^ ICDs successfully treat life-threatening VAs in this population.^8,13^ However, appropriate patient selection is important, as difficult device placement and device-related complications are common among patients with CHD.^14^ Among this heterogeneous group, patients with complex CHD, such as (1) tetralogy of Fallot (TOF); (2) transposition of the great arteries (TGA); or (3) single-ventricle physiology, are at highest risk for SCD.^15^ Risk factors associated with SCD seem to differ among these high-risk substrates, indicating that different risk stratification schemes are necessary for individual congenital defects.^16^ Despite varying substrate-specific SCD risk factors, the current guidelines for ICD use in patients with CHD address the population as a whole.

### Risk stratification

#### Tetralogy of Fallot.

TOF is the most common cyanotic congenital heart defect, occurring in four to five of 10,000 live births.^17^ It is characterized by right ventricular outflow tract obstruction, intraventricular communication, right ventricular hypertrophy, and overriding aorta with displacement towards the right heart. Contemporary methods for surgical repair have improved survival into adulthood; however, SCD due to late-onset malignant VAs has become a leading cause of mortality in this population.^18^ The incidence of sustained ventricular tachycardia (VT) and SCD 35 years after surgical correction is around 12% and 8%, respectively.^18^ Patients with TOF are the most common recipients of ICDs among patients with CHD.^19^

Studies aimed at identifying predictors of SCD in patients with TOF have yielded numerous potential risk factors based on surgical history, clinical parameters, electrocardiographic and electrophysiological metrics, and functional parameters.^15^ The patient factors that are most strongly associated with increased SCD risk are summarized in **Table 1**. Other suggested risk factors that may not be as strongly associated with SCD include older age (after the first decade of life) at the time of complete repair, a history of syncope or rapid palpitations, extensive right ventricular fibrosis on cardiac magnetic resonance imaging (MRI) scan, elevated left ventricular end diastolic pressure, non-sustained VT on ambulatory cardiac monitor, and atrial arrhythmias.^16,18,20–23^ The presence of a single risk predictor portends only moderate risk of SCD.^20^ In patients with moderate risk (ie, the presence of a single risk factor, symptoms suggestive of VAs without ambulatory monitor documentation), inducible VA at electrophysiology study (EPS) is considered to be predictive of clinical malignant arrhythmias. EPS does not, however, predict adverse events in asymptomatic patients without risk factors.^24^ Weighted risk stratification calculators have not yet been prospectively validated.

#### Transposition of the great arteries. 

TGA accounts for 5% to 7% of all congenital heart defects. Surgical intervention for dextro-TGA (D-TGA) has evolved significantly over the last 80 years. The Senning and Mustard procedures, involving atrial baffling to divert blood to the appropriate ventricle, were common in the 1950s to 1980s. These surgeries, also termed atrial switch procedures, result in the morphologic right ventricle as the systemic ventricle, and are subsequently complicated by high rates of systemic ventricular failure due to prolonged exposure to systemic pressures. In the 1980s, surgical interventions targeting switching of the great arteries with restoration of a systemic left ventricle became common. Unlike D-TGA, levoTGA (L-TGA), or congenitally corrected TGA, does not require surgical intervention in childhood, but is associated with failure of the systemic right ventricle later in life.^16^ VAs are the leading cause of death in patients who have undergone the Mustard or Senning procedure. The incidence of SCD in this group is estimated to be 2% to 15%.^25^ VAs following an atrial switch procedure are thought to occur due to systemic ventricular dysfunction and associated mechanoelectrical interactions.^26^ It has also been hypothesized that myocardial ischemia related to abnormal coronary blood flow, hypertrophic remodeling of the systemic right ventricle, and rapid heart rates due to sinus or atrial tachycardia could be a common mechanism for malignant VAs, leading to SCD following the Mustard or Senning procedure.^27^

Unfortunately, there is a paucity of data identifying risk factors for SCD in patients with TGA. Risk factors identified through observational studies are summarized in **Table 1**.^16,25^ Inducible VAs at EPS and high ventricularectopy burden on ambulatory cardiac monitoring have not been associated with SCD in patients with TGA in studies to date.^25,28^ Additionally, risk factors for malignant arrhythmias in the L-TGA population have been poorly studied to date, although systemic right ventricular dysfunction is accepted as a predictor of adverse outcomes and SCD.^29^

#### Single ventricle.

Single-ventricle physiology can be caused by a number of congenital cardiac abnormalities, including hypoplastic left heart syndrome, tricuspid valve atresia, pulmonary valve atresia with intact ventricular septum, and double-inlet left ventricle. These congenital anomalies are rare and are estimated to affect four to eight of 10,000 live births.^30^ Staged surgical procedures during childhood are required, often resulting in Fontan circulation, in which deoxygenated blood is shunted from systemic venous circulation to pulmonary arterial circulation. Despite the completion of surgical correction, affected patients experience high rates of morbidity, including atrial arrhythmias, heart failure, and thromboembolic events.^31^ Patients with single-ventricle physiology and Fontan circulation have the highest mortality rates among those with CHD.^31^

In patients who survive the initial series of corrective surgeries, SCD due to malignant VAs is a common cause of mortality in patients with single-ventricle physiology, following ventricular failure.^32^ Owing to a lack of data identifying risk factors for VAs in this population, proposed predictors of SCD are based on expert opinion. The generally accepted risk factors are summarized in **Table 1**.^15,16^

### Recommendations for implantable cardioverter-defibrillator use

Although distinct risk factor profiles have been identified for each high-risk congenital substrate, current American College of Cardiology/American Heart Association (ACC/AHA) recommendations for ICD implantation in the CHD population address the group as a whole, irrespective of substrate type. This is largely due to a lack of randomized clinical trials assessing primary prevention ICD use in this heterogeneous patient population. Guidelines for secondary prevention ICDs are well defined. ICD implantation is indicated in survivors of cardiac arrest due to ventricular fibrillation (VF) or hemodynamically unstable VT when reversible causes have been excluded [class I, level of evidence (LOE) B].^33^ ICD use is also indicated in patients with spontaneous, symptomatic sustained VT (class I, LOE C).^33^ High rates of appropriate shocks have been observed following ICD placement for secondary prevention in patients with complex CHD.^7,15^ Guidelines-based indications for primary prevention device use are less clear, with only one recommendation currently in place. ICD implantation may be considered for patients with recurrent syncope in the setting of complex congenital heart and advanced systemic ventricular dysfunction when invasive and non-invasive evaluation are unrevealing (class IIb, LOE C).^33^ Two ACC/AHA guidelines are currently being developed that will likely provide additional recommendations in ICD use in these patients: one addresses the adult CHD population, while the other focuses on the management of patients with VAs.

Though official guidelines thoroughly addressing primary prevention ICD use have not yet been published, the Pediatric and Congenital Electrophysiology Society and the Heart Rhythm Society (PACES/ HRS) have released a joint expert consensus statement on the recognition and management of arrhythmias in adults with CHD that provides additional recommendations to aid in clinical decision-making processes in patients with CHD. The PACES/HRS expert consensus statement states that an ICD is indicated in adult CHD patients with biventricular physiology and systemic left ventricular ejection fraction ≤ 35% with New York Heart Association (NYHA) class II or class III symptoms (class I, LOE B).^7^ This recommendation is adapted from ACC/AHA guidelines for the use of ICDs in patients with non-ischemic cardiomyopathy, and is not intended to be applied in patients with single-ventricle physiology. Additional substrate-specific criteria for primary prevention ICD use are also offered through expert opinion based on available observational studies. ICD implantation is reasonable in adults with TOF and more than one risk factor for SCD. Relevant risk factors based on consensus recommendations include non-sustained VT, left ventricular dysfunction, QRS duration ≥ 180 ms, extensive right ventricular scarring on cardiac MRI and VT inducible at EPS (class IIb, LOE B).^7^ An ICD may also be reasonable in adults with a single or systemic right ventricular ejection fraction < 35%. This recommendation is strengthened in the presence of additional risk factors such as nonsustained VAs, unexplained syncope, NYHA function class II or class III symptoms, or QRS duration ≥ 140 ms (class IIb, LOE C). An ICD may also be considered in adults with CHD with unexplained syncope in the setting of hemodynamically significant sustained VT or VF inducible at EPS (class IIb, LOE C).^7^ Finally, an ICD may be considered in patients with complex CHD and unexplained syncope when there is a high clinical suspicion of malignant VA (class IIb, LOE C).^7^ **Table 2** presents a summary of PACES/HRS recommendations for ICD therapy in adults with CHD.^7^

ICD implantation in patients with CHD can present unique procedural challenges. Some of these challenges relate to difficult vascular access and lead placement as well as difficult or impossible access to the desired cardiac chambers due to venous obstruction, conduits, baffles, or total cavopulmonary connection.^15^ Furthermore, in the setting of shunt lesions with right to left flow, transvenous devices are avoided due to a risk of embolic events. For patients in whom transvenous ICD placement is deemed impossible or unsafe, the subcutaneous ICD has emerged as an effective and safe alternative.^34^ As is true in patients without CHD, screening to determine eligibility for the subcutaneous ICD is required prior to implantation in this population. Although it may be perceived that patients with CHD would have lower rates of eligibility for subcutaneous ICD use due to the presence of complex anatomic abnormalities, CHD patients actually have similar rates of eligibility based on screenings compared with those with structurally normal hearts.^35^ It should be kept in mind that patients with bradycardia or those who are expected to develop bradycardia requiring pacing should not be considered for subcutaneous ICD implantation. Epicardial ICDs, which require surgical thoracotomy, are used frequently in the pediatric population, but are implanted less commonly in adults with CHD.

In addition, compared with those without CHD, patients with complex CHD are at a higher risk of ICD implantation-related complication, as well as long-term devicerelated complications such as thromboembolic events, endocarditis, venous occlusion, lead failure, and high rates of inappropriate shocks due to supraventricular arrhythmias.^14,19,36–38^ In all CHD patients, the risks and benefits of ICD implantation should be weighed carefully, with an emphasis placed on shared decision-making. As with other patients who receive a primary prevention ICD, programming parameters should be optimized to avoid inappropriate shocks through long detection times and high detection rates.^39–41^ Prospective clinical trials are necessary to better define evidence-based indications for substrate-specific primary prevention ICD use.

## Inherited arrhythmia syndromes

Cardiac channelopathies, caused by mutations in genes encoding ion channels or channel-related proteins, are a diverse group of inherited conditions that lead to an increased risk of SCD in patients with structurally normal hearts. Over the last three decades, the identification of genes associated with inherited channelopathies has improved both the diagnosis and clinical management of these conditions.^42^ Clinical manifestations can range from asymptomatic carriers to malignant VAs and SCD.^43^ This heterogeneity of disease expression poses significant challenges to appropriate risk stratifications for SCD and patient selection for ICD use. Current disease-specific recommendations are largely based on expert opinion and small observational studies.

### Long QT syndrome

Long QT syndrome (LQTS) is an inherited disorder of ventricular myocardial repolarization characterized by an abnormally long QT interval on the electrocardiogram (ECG) and an increased risk of malignant VAs. It is typically inherited in an autosomal dominant pattern, and affects one out of every 2,000 live births.^44^ The first genetic mutations responsible for the disease were discovered in 1995, with at least 13 others having been found since that time. LQTS can be caused by mutations in genes encoding potassium channel proteins, calcium channel-related proteins, sodium channel proteins, and membrane adaptor proteins.^45,46^ Of those discovered, three genetic mutations (*KCNQ1*, *KCNH2*, and *SCN5A*) account for the most common genotypes.^46^

In the absence of a secondary cause for QT interval prolongation, a clinical diagnosis of congenital LQTS can be made based on a weighted scoring system that incorporates patient age, ECG findings, medical and family history, and symptoms. This scoring system, called the Schwartz score, is summarized in **Table 3**.^47^ Points are additive, with a score of ≥ 3.5 suggestive of the diagnosis. LQTS can also be diagnosed in the following scenarios: unequivocally pathogenic mutation, QT interval corrected for ≥ 500 ms in repeated 12-lead ECGs, and/or QT interval corrected for heart rate 480 ms to 499 ms in the setting of unexplained syncope.^46^ Heart rate and QT interval changes during exercise testing and early recovery can be used to support a presumed diagnosis of LQTS or to identify asymptomatic relatives who are more likely to have pathogenic gene mutations.^48,49^

Genetic testing should be pursued in all patients who are diagnosed with LQTS, as the genotype–phenotype correlation is significant.^50^ The three most common genotypepositive phenotypes (LQT1, LQT2, and LQT3) are associated with distinct triggers for VAs and SCD. Patients with LQT1 are most likely to experience arrhythmic events during physical exertion and emotional stress, while SCD in LQT2 is typically associated with sudden auditory stimulation. Patients with LQT3, in contrast, are most likely to have SCD at night while sleeping.^51^ The most common presenting symptom in patients with LQTS is syncope; however, SCD is rarely the first clinical manifestation.^46^

#### Risk stratification.

LQTS has variable clinical manifestations, and not all patients diagnosed with the disease experience life-threatening arrhythmias. Risk stratification aimed at identifying those at highest risk for SCD is therefore important.

Notably, the location and type of genetic mutation can affect prognosis. In patients with LQT1, missense and transmembrane mutations lead to longer QT intervals and a higher risk of SCD, while mutations in the C-terminal region are typically associated with a lowrisk phenotype.^52,53^ Mutations in the cytoplasmic loops of *KCNQ1* and pore region of *KCNH2* are associated with higher rates of SCD.^54,55^ Jervelle and Lange-Nielsen syndrome, which is associated with sensorineural deafness, is a particularly high-risk variant caused by mutations in *KCNQ1* or *KCNE1*.^56^ With continued technological advances, the ability to utilize patient-specific genetic information to stratify risk will become more robust.

Clinical characteristics including patient and historical factors, as well as ECG findings, can also be used to identify high-risk patients. A history of cardiac arrest, syncope, or sustained VT, especially when occurring at a younger age and/or while on β-blocker therapy, are historical high-risk features.^46,57^ Men with LTQ1 who remain asymptomatic through the first decades of life are unlikely to develop malignant arrhythmias in adulthood, while women, especially those with LQT2, remain at high risk for VAs even when asymptomatic through middleaged adulthood.^46^ High-risk ECG findings include QT interval corrected for heart rate ≥ 550 ms and the presence of T-wave alternans.^46^ Concealed mutation-positive patients who have a normal QT interval are at low risk for arrhythmic events.^58^ A family history of SCD is not associated with increased risk.^59^

#### Recommendations for implantable cardioverter-defibrillator use.

Appropriate patient selection for ICD implantation is important, as those with LQTS who receive an ICD have high rates of lifetime complications and inappropriate shocks.^60^ A combination of ACC/ AHA guidelines as well as expert consensus recommendations can be used to guide ICD use in patients with LQTS. ICD implantation is recommended in LQTS patients who are survivors of cardiac arrest (class I, LOE A). ICD implantation is also considered useful in patients with LQTS who experience recurrent syncope or VAs despite β-blocker use (class IIa, LOE B). ICD use may also be considered for prophylaxis of SCD in patients at high risk for cardiac arrest (class IIb, LOE B). Patients with multiple high-risk features are most likely to receive benefit from primary prevention ICD use.^46^ Currently, ICD use is not indicated in completely asymptomatic patients, irrespective of genetic profile.^61^ Whether or not the imminent guidelines for the management of patients with VAs will refine these recommendations remains to be determined.

If an ICD is implanted, programming techniques to avoid inappropriate shocks should be emphasized. Given that *torsades de pointes*, a typically fast and disorganized VA, is the most common rhythm associated with SCD in patients with LQTS, prolonged detection time and high detection rates should be used. Additionally, backup pacing at 70 bpm to 80 bpm can also be used to avoid pausedependent *torsades de pointes*.

### Brugada syndrome

Brugada syndrome (BrS) is inherited through an autosomal dominant pattern, and is diagnosed by characteristic ECG findings. The epidemiology of the disease is not well understood, but its prevalence is estimated to be around five in 10,000 individuals worldwide; research has suggested, however, that it is most common in patients of southeast Asian descent.^62^ More than 12 responsible genes have been identified; around one-third of patients with diagnostic ECG findings have an identifiable pathogenic mutation.^63,64^

There are three ECG morphologies associated with BrS. Type 1 morphology is characterized by the presence of at least 2 mm of coved ST segment elevation in at least one right precordial lead (V1–V4). Type 2 Brugada pattern also demonstrates the presence of least 2 mm of S segment elevation in at least one right precordial lead, but the ST segments in this type of BrS have a saddleback appearance, and are typically followed by positive or biphasic T-waves. Type 3 Brugada pattern has either coved or saddleback ST segment elevation in the same leads, but the magnitude of elevation is less than 2 mm. BrS is diagnosed in the setting of spontaneous type 1 pattern on ECG. The syndrome can also be diagnosed when type 1 pattern develops following provocative drug testing with class I antiarrhythmics (or other provocative factors such as fever, cocaine use, or excessive alcohol use) and at least one of the following clinical characteristics: documented VT or polymorphic VT, a family history of SCD before age of 45 years, spontaneous type 1 Brugada pattern in family members, and/or recurrent syncope or nocturnal agonal respirations. Of note, types 2 and 3 Brugada patterns are not diagnostic of BrS, but provocative drug testing should be pursued in these patients.^46^

#### Risk stratification.

BrS patients at highest risk of SCD include those with a prior history of cardiac arrest or with hemodynamically significant sustained VAs. Patients who have survived cardiac arrest have high rates of recurrent life-threatening arrhythmias irrespective of other risk factors.^65^ Additionally, recurrent syncope in the setting of spontaneous type 1 Brugada pattern on ECG portends a high risk of arrhythmic events.^65,66^ The significance of predictors in asymptomatic patients is less clear, but identified risk factors include spontaneous type 1 ECG morphology, QRS complex fragmentation, male gender, and spontaneous atrial fibrillation.^46,67^ Unlike in the case of LQTS, specific genetic mutations causing BrS do not appear to be prognostically important.^64^ Additionally, the role of EPS to assess inducibility is controversial in this population, with conflicting data regarding its value in predicting clinical VAs.^66,68^

#### Recommendations for implantable cardioverter-defibrillator use.

ICD implantation is an effective treatment for the prevention of SCD in patients with BrS. ACC/AHA guidelines state that an ICD is indicated for survivors of cardiac arrest (class I, LOE C). An ICD can also be considered in patients with spontaneous type 1 Brugada morphology who have had syncope without a clear cause (class IIa, LOE C). ICD use is also reasonable for patients with BrS who have had documented VT (class IIa, LOE C).^61^ The mention of other patient characteristics identified as high-risk features through small observation studies has not yet been included in ACC/AHA or expert consensus guidelines.

### Catecholaminergic polymorphic ventricular tachycardia

Catecholaminergic polymorphic ventricular tachycardia (CPVT) is a rare proarrhythmic disorder characterized by adrenergically induced bidirectional or poly morphic VT and SCD.^42^ Its prevalence is accepted to be one out of 10,000 individuals, though this estimate is not derived from any systematic assessment of the general population.^69^

Without a family history, CPVT is difficult to diagnose in asymptomatic patients, as their resting ECGs are typically without abnormalities. Two genetic variants exist: an autosomal dominant form caused by mutations in the gene coding for the cardiac ryanodine receptor (*RYR2*), known as CPVT1, and a much less common autosomal recessive form caused by mutations in the gene coding for calsequestrin (*CASQ2*), known as CPVT2.^70^ At least one of these mutations is identified in 60% of cases.^64^ CPVT is diagnosed in patients with structurally normal hearts, normal resting ECGs, and unexplained exercise- or catecholamine-induced bidirectional or polymorphic premature ventricular complexes or VT. Coronary artery disease must be ruled out in patients older than 40 years of age. The disease can also be diagnosed in asymptomatic patients with a pathogenic mutation.^46^

#### Risk stratification.

Risk stratification for CPVT is based primarily on symptom presentation and response to exercise stress testing. Cardiac arrest as the presenting symptom, as opposed to syncope, is associated with high rates of recurrent malignant arrhythmias.^71^ The diagnosis of CPVT during childhood also predicts adverse outcomes. Complex ectopy during exercise predicts increased rates of SCD and clinical VAs.^71^

Currently, genetic analysis does not contribute to risk stratification.^61^ Evidence is emerging, however, in support of genotype–phenotype correlation. Mutations in the C-terminal channel-forming domain of the RYR2 gene have been associated with increased odds of development of VA compared with mutations affecting the N-terminal domain.^72^ Further development of a risk stratification scheme has been difficult due to the low prevalence of disease.^61^

Inducible VA with EPS is not predictive of adverse outcomes in CPVT, and is therefore not recommended.^46^

#### Recommendations for implantable cardioverter-defibrillator use.

As with other cardiac channelopathies, recommendations for ICD use in patients with CPVT are largely based on expert opinion and the results of observational studies. ICD implantation is recommended, in conjunction with initiation of β-blocker therapy, in survivors of cardiac arrest who are diagnosed with CPVT (class I, LOE C). In general, CPVT is controllable with lifestyle modification and β-blocker therapy. In patients who have not experienced cardiac arrest, ICD therapy is only recommended in the setting of recurrent syncope or documented bidirectional or polymorphic VT, despite β-blocker use (class IIa, LOE C).^61^ ICD implantation is not recommended in asymptomatic patients. When ICD therapy is used, device programming should include long delays before shock delivery and high detection rates, as painful, inappropriate shocks or shocks for hemodynamically stable or self-terminating VAs can increase sympathetic tone and lead to episodes of life-threatening arrhythmia storm.^46^ It should be noted that ICDs alone are not sufficient treatment for patients with CPVT. To avoid electrical storm, the treatment of these individuals should be focused on the prevention of VA through the use of maximally tolerated doses of β-blocker or calcium channel blocker therapy, with or without flecainide.

### Short QT syndrome

Short QT syndrome (SQTS) is an extremely rare inherited cardiac channelopathy characterized by an abnormally short repolarization period and a corresponding short QT interval.^61^ The diagnosis of SQTS by ECG parameters is the subject of ongoing debate. Based on expert opinion, current diagnostic criteria include the presence of a QTc ≤ 330 ms, or a QTc ≤ 360 ms and at least one of the following: pathogenic mutation, family history of SQTS, family history of SCD at ≤ 40 years of age, and prior VA in the absence of structural heart disease.^46^ Pathologic genetic mutations include gain-of-function mutations of *KCNH2*, *KCNQ1*, and *KCNJ2*.^73–75^

The management of patients with SQTS and risk stratification for appropriate ICD use are not well defined. Patients with SQTS with prior episodes of sustained VA are at high risk of recurrent arrhythmia and SCD.^76^ ICD implantation is therefore recommended in survivors of cardiac arrest and/or in those with documented spontaneous sustained VA with or without syncope (class I, LOE not defined).^46^ Based on expert opinion, it may also be appropriate to consider ICD use in asymptomatic patients with a diagnosis of SQTS and a family history of SCD (class IIb, LOE not defined).^46^ It should be noted that tall and peaked T-waves are typically associated with the short QT interval seen in SQTS, causing high rates of T-wave oversensing and inappropriate ICD shocks. Appropriate programmed detection algorithms in ICDs can help to minimize the risk of this complication.^77^

### Early repolarization syndrome

Early repolarization (ER) is a common ECG pattern characterized by J-point and ST-segment elevation in two or more contiguous leads. Although considered a benign finding when present in the anterior precordial leads, the presence of ER in the inferior and/or lateral leads has recently been associated with an increased risk of SCD, leading to the identification of ER syndrome (ERS).^46,78^ ERS is diagnosed in patients with an ER pattern on ECG, particularly in the inferior and/or lateral leads, who have survived a documented episode of VF or polymorphic VT without the presence of other known cardiac disease.^46^

Based on the findings from a limited number of casecontrol studies, it is suggested that patients with an ER pattern who are at highest risk of SCD include those with slurred or notched J-point elevations of ≥ 2 mV, a highamplitude J-point elevation, an ER pattern in the inferior and/or lateral leads, and a horizontal or descending ST segment following J-point elevation.^78–81^ The guidelines for ICD use in these patients are based on expert opinion. ICD use is recommended in patients with ERS who have survived an episode of cardiac arrest (class I, LOE not defined). ICD implantation can also be considered in family members of patients with ERS who have a history of syncope in the setting of ST-segment elevation > 1 mm in two or more inferior or lateral leads (class IIb, LOE not defined). Finally, ICD use can be considered in asymptomatic patients with a family history of unexplained sudden death during childhood in the presence of a high-risk ECG pattern (class IIb, LOE not defined). ICD use is not recommended, however, in asymptomatic patients who do not have high-risk ECG findings.^46^

## Conclusions

CHD and inherited arrhythmia syndromes increase the risk of malignant VAs and SCD in some patients. Although little controversy exists surrounding the use of secondary prevention ICDs in these individuals, risk stratification to identify patients who should undergo primary prevention ICD implantation remains poorly defined. Most guidelines outlining the recommended indications for primary prevention ICD use for patients with CHD and inherited arrhythmia syndromes are based on case reports, observational studies, and expert opinion. Further investigation is needed to establish appropriate risk stratification schema capable of identifying patients within these growing populations who are most likely to benefit from primary prevention ICD use.

## Figures and Tables

**Table 1:** Risk Factors for SCD in Patients with CHD

**Tetralogy of Fallot**	
•	Transannular patch^18^
•	Severe pulmonary regurgitation^18^
•	Right ventricular hypertrophy^20^
•	Right ventricular dilation^22^
•	Right ventricular systolic dysfunction^20^
•	Left ventricular systolic dysfunction^82^
•	QRS duration ≥ 180 ms^18,22^
**Transposition of the Great Arteries**	
•	Longer period of time since surgical correction^83^
•	Depressed systemic right ventricular dysfunction^83^
•	QRS duration > 140 ms^83^
•	Atrial arrhythmia^25^
•	History of syncope or rapid palpitations^25^
Single Ventricle^*^	
•	Longer period of time since surgical correction
•	History of syncope or rapid palpitations
•	Atrial arrhythmia
•	Depressed single-ventricle function

*Based on expert opinion.^15,16^

SCD: sudden cardiac death; CHD: congenital heart disease.

**Table 2:** PACES/HRS Recommendations for ICD Therapy in Adults with CHD^7^

Class I	•	ICD therapy is indicated in adults with CHD who are survivors of cardiac arrest due to ventricular fibrillation of hemodynamically unstable ventricular tachycardia after evaluation to define the cause of the event and exclude any completely reversible etiology (LOE B).
	•	ICD therapy is indicated in adults with CHD and spontaneous sustained ventricular tachycardia who have undergone hemodynamic and electrophysiologic evaluation (LOE B).
	•	ICD therapy is indicated in adults with CHD and a systemic left ventricular ejection fraction ≤ 35%, biventricular physiology and NYHA class II or III symptoms (LOE B).
Class IIa	•	ICD therapy is reasonable in selected adults with tetralogy of Fallot and multiple risk factors for sudden cardiac death, such as left ventricular systolic or diastolic dysfunction, non-sustained ventricular tachycardia, QRS duration ≥ 180 ms, extensive right ventricular scarring, or inducible sustained ventricular tachycardia at EPS (LOE B).
Class IIb	•	ICD therapy may be reasonable in adults with a single or systemic right ventricular ejection fraction < 35%, particularly in the presence of additional risk factors such as complex ventricular arrhythmias, unexplained syncope, NYHA functional class II or III symptoms, QRS duration ≥ 140 ms, or severe systemic atrioventricular valve regurgitation (LOE C).
	•	ICD therapy may be considered in adults with CHD and a systemic ventricular ejection fraction < 35% in the absence of overt symptoms (NYHA class I) or other known risk factors (LOE C).
	•	ICD therapy may be considered in adults with CHD and syncope of unknown origin with hemodynamically significant sustained ventricular tachycardia or fibrillation inducible at EPS (LOE B).
	•	ID therapy may be considered for non-hospitalized adults with CHD awaiting heart transplantation (LOE C).
	•	ICD therapy may be considered for adults with syncope and moderate or complex CHD in whom there is a high clinical suspicion of ventricular arrhythmia and in whom thorough invasive and non-invasive investigations have failed to define a cause (LOE C).
Class III	•	All class III recommendations in current ACC/AHA/HRS guidelines apply to adults with CHD (LOE C).
	•	Adults with CHD and advanced pulmonary vascular disease (LOE B).
	•	Endocardial leads are generally avoided in adults with CHD and intracardiac shunts (LOE B).

PACES/HRS: Pediatric and Congenital Electrophysiology Society/Heart Rhythm Society; ICD: implantable cardioverter-defibrillator; CHD: congenital heart disease; LOE: level of evidence; EPS: electrophysiology study; NYHA: New York Heart Association; ACC/AHA/HRS: American College of Cardiology/American Heart Association/Heart Rhythm Society.

**Table 3:** The Schwartz Score for the Diagnosis of LQTS^47^

QTc at rest	
≥ 480 ms	3 points
460–470 ms	2 points
450–460 ms	1 point
QTc at the fourth minute of recovery following exercise stress test	
≥ 480 ms	1 point
Torsades de pointes*	2 points
T-wave alternans	1 point
Notched T-wave in > three leads	1 point
Syncope*	
With stress	2 points
Without stress	1 point
Family member with LQTS**	1 point
Unexplained SCD in an immediate family member < 30 years of age**	0.5 points

*Points for torsades de pointes and syncope are mutually exclusive.

**The same family member cannot be counted for both criteria.

LQTS: long QT syndrome; SCD: sudden cardiac death.

## References

[1] Mozaffarian D, Benjamin EJ, Go AS (2015). Heart disease and stroke statistics—2015 update: a report from the American Heart Association. Circulation..

[2] Moss AJ, Zareba W, Hall WJ (2002). Prophylactic implantation of a defibrillator in patients with myocardial infarction and reduced ejection fraction. N Engl J Med..

[3] Bardy GH, Lee KL, Mark DB (2005). Amiodarone or an implantable cardioverter-defibrillator for congestive heart failure. N Engl J Med..

[4] Russo AM, Stainback RF, Bailey SR (2013). ACCF/HRS/AHA/ASE/HFSA/SCAI/SCCT/SCMR 2013 appropriate use criteria for implantable cardioverter-defibrillators and cardiac resynchronization therapy: a report of the American College of Cardiology Foundation appropriate use criteria task force, Heart Rhythm Society, American Heart Association, American Society of Echocardiography, Heart Failure Society of America, Society for Cardiovascular Angiography and Interventions, Society of Cardiovascular Computed Tomography, and Society for Cardiovascular Magnetic Resonance. Heart Rhythm..

[5] Oechslin EN, Harrison DA, Connelly MS, Webb GD, Siu SC (2000). Mode of death in adults with congenital heart disease. Am J Cardiol..

[6] Engelings CC, Helm PC, Abdul-Khaliq H (2016). Cause of death in adults with congenital heart disease—An analysis of the German National Register for Congenital Heart Defects. Int J Cardiol..

[7] Khairy P, Van Hare GF, Balaji S (2014). PACES/HRS expert consensus statement on the recognition and management of arrhythmias in adult congenital heart disease: developed in partnership between the Pediatric and Congenital Electrophysiology Society (PACES) and the Heart Rhythm Society (HRS). Endorsed by the governing bodies of PACES, HRS, the American College of Cardiology (ACC), the American Heart Association (AHA), the European Heart Rhythm Association (EHRA), the Canadian Heart Rhythm Society (CHRS), and the International Society for Adult Congenital Heart Disease (ISACHD). Can J Cardiol..

[8] Koyak Z, de Groot JR, Van Gelder IC (2012). Implantable cardioverter defibrillator therapy in adults with congenital heart disease: who is at risk of shocks?. Circ Arrhythm Electrophysiol..

[9] Zareba W, Moss AJ, Daubert JP, Hall WJ, Robinson JL, Andrews M (2003). Implantable cardioverter defibrillator in highrisk long QT syndrome patients. J Cardiovasc Electrophysiol..

[10] Maron BJ, Shen WK, Link MS (2000). Efficacy of implanta-ble cardioverter-defibrillators for the prevention of sudden death in patients with hypertrophic cardiomyopathy. N Engl J Med..

[11] Sears SF, St Amant JB, Zeigler V (2009). Psychosocial considerations for children and young adolescents with implantable cardioverter defibrillators: an update. Pacing Clin Electrophysiol..

[12] Avila P, Mercier LA, Dore A (2014). Adult congenital heart disease: a growing epidemic. Can J Cardiol..

[13] Khairy P, Harris L, Landzberg MJ (2008). Implantable cardioverter-defibrillators in tetralogy of Fallot. Circulation..

[14] Santharam S, Hudsmith L, Thorne S, Clift P, Marshall H, De Bono J (2017). Long-term follow-up of implantable cardioverter-defibrillators in adult congenital heart disease patients: indications and outcomes. Europace..

[15] Avila P, Chaix MA, Mondésert B, Khairy P (2017). Sudden cardiac death in adult congenital heart disease. Card Electrophysiol Clin..

[16] Walsh EP (2014). Sudden death in adult congenital heart disease: risk stratification in 2014. Heart Rhythm..

[17] Centers for Disease Control and Prevention (CDC) (2006). Improved national prevalence estimates for 18 selected major birth defects—United States, 1999–2001. MMWR Morb Mortal Wkly Rep..

[18] Gatzoulis MA, Balaji S, Webber SA (2000). Risk fac-tors for arrhythmia and sudden cardiac death late after repair of tetralogy of Fallot: a multicentre study. Lancet..

[19] Vehmeijer JT, Brouwer TF, Limpens J (2016). Implantable cardioverter-defibrillators in adults with congenital heart disease: a systematic review and meta-analysis. Eur Heart J..

[20] Valente AM, Gauvreau K, Assenza GE (2014). Contemporary predictors of death and sustained ventricular tachycardia in patients with repaired tetralogy of Fallot enrolled in the INDICATOR cohort. Heart..

[21] Daliento L, Rizzoli G, Menti L (1999). Accuracy of electro-cardiographic and echocardiographic indices in predicting life threatening ventricular arrhythmias in patients operated for tetralogy of Fallot. Heart..

[22] Gatzoulis MA, Till JA, Somerville J, Redington AN (1995). Mechanoelectrical interaction in tetralogy of Fallot. QRS prolongation relates to right ventricular size and predicts malignant ventricular arrhythmias and sudden death. Circulation..

[23] Park SJ, On YK, Kim JS (2012). Relation of fragmented QRS complex to right ventricular fibrosis detected by late gadolinium enhancement cardiac magnetic resonance in adults with repaired tetralogy of fallot. Am J Cardiol..

[24] Khairy P (2007). Programmed ventricular stimulation for risk stratification in patients with tetralogy of Fallot: a Bayesian perspective. Nat Clin Pract Cardiovasc Med..

[25] Kammeraad JA, van Deurzen CH, Sreeram N (2004). Predictors of sudden cardiac death after Mustard or Senning repair for transposition of the great arteries. J Am Coll Cardiol..

[26] Sathananthan G, Harris L, Nair K (2017). Ventricular arrhythmias in adult congenital heart disease: mechanisms, diagnosis, and clinical aspects. Card Electrophysiol Clin..

[27] Khairy P (2017). Sudden cardiac death in transposition of the great arteries with a Mustard or Senning baffle: the myocardial ischemia hypothesis. Curr Opin Cardiol..

[28] Khairy P, Harris L, Landzberg MJ (2008). Sudden death and defibrillators in transposition of the great arteries with intra-atrial baffles: a multicenter study. Circ Arrhythm Electrophysiol..

[29] Connelly MS, Liu PP, Williams WG, Webb GD, Robertson P, McLaughlin PR (1996). Congenitally corrected transposition of the great arteries in the adult: functional status and complications. J Am Coll Cardiol..

[30] O’Leary PW (2002). Prevalence, clinical presentation and natural history of patients with single ventricle. Prog Pediatr Cardiol..

[31] Khairy P, Fernandes SM, Mayer JE Jr (2008). Long-term survival, modes of death, and predictors of mortality in patients with Fontan surgery. Circulation..

[32] Alsaied T, Bokma JP, Engel ME (2017). Factors associated with long-term mortality after Fontan procedures: a systematic review. Heart..

[33] Epstein AE, DiMarco JP, Ellenbogen KA (2008). ACC/AHA/HRS 2008 Guidelines for Device-Based Therapy of Cardiac Rhythm Abnormalities: a report of the American College of Cardiology/American Heart Association Task Force on Practice Guidelines (Writing Committee to Revise the ACC/ AHA/NASPE 2002 Guideline Update for Implantation of Cardiac Pacemakers and Antiarrhythmia Devices) developed in collaboration with the American Association for Thoracic Surgery and Society of Thoracic Surgeons. J Am Coll Cardiol..

[34] Moore JP, Mondésert B, Lloyd MS (2016). Clinical experience with the subcutaneous implantable cardioverter- defibrillator in adults with congenital heart disease. Circ Arrhythm Electrophysiol..

[35] Zeb M, Curzen N, Veldtman G (2015). Potential eligibility of congenital heart disease patients for subcutaneous implantable cardioverter-defibrillator based on surface electrocardiogram mapping. Europace..

[36] Walsh EP (2008). Practical aspects of implantable defibrillator therapy in patients with congenital heart disease. Pacing Clin Electrophysiol..

[37] Khairy P, Landzberg MJ, Gatzoulis MA (2006). Transvenous pacing leads and systemic thromboemboli in patients with intracardiac shunts: a multicenter study. Circulation..

[38] Hayward RM, Dewland TA, Moyers B (2015). Device com-plications in adult congenital heart disease. Heart Rhythm..

[39] Khairy P, Mansour F (2011). Implantable cardioverter-defibrillators in congenital heart disease: 10 programming tips. Heart Rhythm..

[40] Moss AJ, Schuger C, Beck CA (2012). Reduction in inappro-priate therapy and mortality through ICD programming. N Engl J Med..

[41] Gasparini M, Lunati MG, Proclemer A (2017). Long detection programming in single chamber defibrillators reduces unnecessary therapies and mortality: the ADVANCE III trial. JACC Clin Electrophysiol..

[42] Mizusawa Y (2016). Recent advances in genetic testing and counseling for inherited arrhythmias. J Arrhythm..

[43] Martin CA, Matthews GD, Huang CL (2012). Sudden cardiac death and inherited channelopathy: the basic electrophysiology of the myocyte and myocardium in ion channel disease. Heart..

[44] Schwartz PJ, Stramba-Badiale M, Crotti L (2009). Prevalence of the congenital long-QT syndrome. Circulation..

[45] Curran ME, Splawski I, Timothy KW, Vincent GM, Green ED, Keating MT (1995). A molecular basis for cardiac arrhythmia: HERG mutations cause long QT syndrome. Cell..

[46] Priori SG, Wilde AA, Horie M (2013). HRS/EHRA/APHRS expert consensus statement on the diagnosis and management of patients with inherited primary arrhythmia s yndromes: document endorsed by HRS, EHRA, and APHRS in May 2013 and by ACCF, AHA, PACES, and AEPC in June 2013. Heart Rhythm..

[47] Schwartz PJ, Crotti L (2011). QTc behavior during exercise and genetic testing for the long-QT syndrome. Circulation..

[48] Sy RW, van der Werf C, Chattha IS (2011). Derivation and validation of a simple exercise-based algorithm for prediction of genetic testing in relatives of LQTS probands. Circulation..

[49] Swan H, Viitasalo M, Piippo K, Laitinen P, Kontula K, Toivonen L (1999). Sinus node function and ventricular repolarization during exercise stress test in long QT syndrome patients with KvLQT1 and HERG potassium channel defects. J Am Coll Cardiol..

[50] Ackerman MJ, Priori SG, Willems S (2011). HRS/EHRA expert consensus statement on the state of genetic testing for the channelopathies and cardiomyopathies: this document was developed as a partnership between the Heart Rhythm Society (HRS) and the European Heart Rhythm Association (EHRA). Europace..

[51] Schwartz PJ, Prior SG, Spazzolini CM (2001). Genotype– phenotype correlation in the long-QT syndrome: gene- specific triggers for life-threatening arrhythmias. Circulation..

[52] Moss AJ, Shimizu W, Wilde AA (2007). Clinical aspects of type-1 long-QT syndrome by location, coding type, and biophysical function of mutations involving the KCNQ1 gene. Circulation..

[53] Donger C, Denjoy I, Berthet M (1997). KVLQT1 C-terminal missense mutation causes a forme fruste long-QT syndrome. Circulation..

[54] Barsheshet A, Goldenberg I, O-Uchi J (2012). Mutations in cytoplasmic loops of the KCNQ1 channel and the risk of life-threatening events: implications for mutation-specific response to beta-blocker therapy in type 1 long-QT syndrome. Circulation..

[55] Shimizu W, Moss AJ, Wilde AA (2009). Genotype–pheno-type aspects of type 2 long QT syndrome. J Am Coll Cardiol..

[56] Schwartz PJ, Spazzolini C, Crotti L (2006). The Jervell and Lange-Nielsen syndrome: natural history, molecular basis, and clinical outcome. Circulation..

[57] Goldenberg I, Moss AJ, Peterson DR (2008). Risk factors for aborted cardiac arrest and sudden cardiac death in children with the congenital long-QT syndrome. Circulation..

[58] Priori SG, Schwartz PJ, Napolitano C (2003). Risk stratification in the long-QT syndrome. N Engl J Med..

[59] Kaufman ES, McNitt S, Moss AJ (2008). Risk of death in the long QT syndrome when a sibling has died. Heart Rhythm..

[60] Schwartz PJ, Spazzolini C, Priori SG (2010). Who are the long-QT syndrome patients who receive an implantable cardioverter-defibrillator and what happens to them?: data from the European Long-QT Syndrome Implantable Cardioverter-Defibrillator (LQTS ICD) Registry. Circulation..

[61] Zipes DP, European Heart Rhythm Association, Heart Rhythm Society (2006). ACC/AHA/ESC 2006 guidelines for management of patients with ventricular arrhythmias and the prevention of sudden cardiac death: a report of the American College of Cardiology/American Heart Association Task Force and the European Society of Cardiology Committee for Practice Guidelines (Writing Committee to Develop Guidelines for Management of Patients With Ventricular Arrhythmias and the Prevention of Sudden Cardiac Death). J Am Coll Cardiol..

[62] Postema PG (2012). About Brugada syndrome and its prevalence. Europace..

[63] Mizusawa Y, Wilde AA (2012). Brugada syndrome. Circ Arrhythm Electrophysiol..

[64] Ackerman MJ, Priori SG, Willems S (2011). HRS/EHRA expert consensus statement on the state of genetic testing for the channelopathies and cardiomyopathies: this document was developed as a partnership between the Heart Rhythm Society (HRS) and the European Heart Rhythm Association (EHRA). Europace..

[65] Brugada J, Brugada R, Antzelevitch C, Towbin J, Nademanee K, Brugada P (2002). Long-term follow-up of individuals with the electrocardiographic pattern of right bundle-branch block and ST-segment elevation in precordial leads V1 to V3. Circulation..

[66] Priori SH, Gasparini M, Napolitano C (2012). Risk stratification in Brugada syndrome: results of the PRELUDE (PRogrammed ELectrical stimUlation preDictive valuE) registry. J Am Coll Cardiol..

[67] Kusano KF, Taniyama M, Nakamura K (2008). Atrial fibrillation in patients with Brugada syndrome relationships of gene mutation, electrophysiology, and clinical backgrounds. J Am Coll Cardiol..

[68] Sieira J, Conte G, Ciconte G (2015). Prognostic value of programmed electrical stimulation in Brugada syndrome: 20 years experience. Circ Arrhythm Electrophysiol..

[69] van der Werf C, Wilde AA (2013). Catecholaminergic polymorphic ventricular tachycardia: from bench to bedside. Heart..

[70] Mohamed U, Napolitano C, Priori SG (2007). Molecular and electrophysiological bases of catecholaminergic polymorphic ventricular tachycardia. J Cardiovasc Electrophysiol..

[71] Hayashi M, Denjoy I, Extramiana F (2009). Incidence and risk factors of arrhythmic events in catecholaminergic polymorphic ventricular tachycardia. Circulation..

[72] van der Werf C, Nederend I, Hofman N (2012). Familial evaluation in catecholaminergic polymorphic ventricular tachycardia: disease penetrance and expression in cardiac ryanodine receptor mutation-carrying relatives. Circ Arrhythm Electrophysiol..

[73] Brugada R, Hong K, Dumaine R (2004). Sudden death associated with short-QT syndrome linked to mutations in HERG. Circulation..

[74] Bellocq C, van Ginneken AC, Bezzina CR (2004). Mutation in the KCNQ1 gene leading to the short QT-interval syndrome. Circulation..

[75] Priori SG, Pandit SV, Rivolta I (2005). A novel form of short QT syndrome (SQT3) is caused by a mutation in the KCNJ2 gene. Circ Res..

[76] Giustetto C, Schimpf R, Mazzanti A (2011). Long-term followup of patients with short QT syndrome. J Am Coll Cardiol..

[77] Wolpert C, Scimpf R, Veltmann C, Giustetto C, Gaita F, Borggrefe M (2005). Clinical characteristics and treatment of short QT syndrome. Expert Rev Cardiovasc Ther..

[78] Haissaguerre M, Derval N, Sacher F (2008). Sudden cardiac arrest associated with early repolarization. N Engl J Med..

[79] Tikkanen JT, Anttonen O, Junttila MJ (2009). Long-term out-come associated with early repolarization on electrocardiography. N Engl J Med..

[80] Derval N, Simpson CS, Birnie DH (2011). Prevalence and characteristics of early repolarization in the CASPER registry: cardiac arrest survivors with preserved ejection fraction registry. J Am Coll Cardiol..

[81] Tikkanen JT, Junttila MJ, Anttonen O (2011). Early repolarization: electrocardiographic phenotypes associated with favorable long-term outcome. Circulation..

[82] Diller GP, Kempny A, Liodakis E (2012). Left ventricular longitudinal function predicts life-threatening ventricular arrhythmia and death in adults with repaired tetralogy of fallot. Circulation..

[83] Schwerzmann M, Salehian O, Harris L (2009). Ventricular arrhythmias and sudden death in adults after a Mustard operation for transposition of the great arteries. Eur Heart J..

